# Gastrointestinal functional disorders can benefit from the use of medical devices made of substances

**DOI:** 10.3389/fdsfr.2023.1119353

**Published:** 2023-02-24

**Authors:** Vincenzo Savarino, Elisa Marabotto, Patrizia Zentilin, Manuele Furnari, Giorgia Bodini, Edoardo Giovanni Giannini, Edoardo Vincenzo Savarino

**Affiliations:** ^1^ Department of Internal Medicine, University of Genoa, Genoa, Italy; ^2^ Department of Surgery, Oncology and Gastroenterology, University of Padua, Padua, Veneto, Italy

**Keywords:** pharmacological agents, reflux hypersensitivity, functional dyspepsia, irritable bowel syndrome, medical devices made of substances

## Abstract

Medical devices made of substances (MDMS) have recently gained great popularity in several specialties of internal medicine, including gastroenterology. In the last decades this discipline has known relevant advances in the cure of severe diseases, such as peptic ulcer, gastroesophageal reflux disease and chronic hepatitis C, thanks to the revolutionary development of new drugs able to act on single receptors changing a particular cell function or blocking microbial and viral replication. However, there are many gastroenterological illnesses that are difficult to treat with traditional medicinal products because of their complex and poorly known pathophysiology, which comprises altered motility, visceral hypersensitivity, gut dysbiosis, intestinal mild inflammation with impaired immune function, increased mucosal permeability and abnormal brain-gut interaction. They are mainly represented by esophageal functional disorders (reflux hypersensitivity, functional heartburn), functional dyspepsia, irritable bowel syndrome, functional constipation and functional diarrhea. Traditional drugs do not provide a definitive resolution of these disorders with a multifactorial pathogenesis and they can benefit from the use of MDMS, which seem to have the ability to act on different factors thanks to the synergistic action of their various components. International medical literature already reports many clinical trials performed with the well-known standards for evaluating their efficacy and safety in a great part of the above-mentioned conditions.

## 1 Introduction

Medical devices made of substances (MDMS) have gained great popularity in last years and have contributed to enlarge the therapeutic armamentarium in many internal medicine disciplines, including gastroenterology. They differ from medicinal products because their action is not characterized by pharmacological, immunological or metabolic means (Bilia et al., 2021). Despite this negative definition, MDMS are intended to have a therapeutic effect due to unknown mechanisms that need to be defined as clearly as possible. This effort requires the combined collaboration of pharmacologists, clinicians and regulators in order to ensure the clinical use of these products within the strictest safety and the objective evaluation of their efficacy (Racchi et al., 2016).

(Racchi et al., 2016) report that the principal action of a MDMS is typically fulfilled by non-pharmacological means, such as physical means, including mechanical action, physical barrier lubrification and support to organs or body functions, or chemical means, such as pH modifications or any other acid-base reactions and chelation. These compounds often may have more than one non-pharmacological mechanism of action concurring to the claimed therapeutic effect. In fact, MDMS have the specificity of being composed of a very high number of molecules, acting in synchrony, in a way that is best represented by the concept of system, that is its effect depends on the inter-actions and inter-relations among each molecule rather than on a simple sum of its components. In other words, the mechanism of action of MDMS is linked to the entire product and not to one selected single component of it.

The Regulation 2017/745, issued by the EU parliament and the Council (Regulation (EU), 2017) states that MDMS need to be absorbed in order to achieve their intended action and this renders them similar to medicinal products, although their mechanism of action is different. Indeed, they do not act on a single pharmacological target that permits to change the function of a given cell, but may equally play an important therapeutic role in the healthcare system because there are still many illnesses with a relative low grade of risk and not responding adequately to traditional drugs. These conditions impair greatly the patients’ quality of life, are frequently shared by many people and their clinical behavior is characterized by a chronic evolution with alternate phases of exacerbation and remission.

The discipline of gastroenterology comprises many disorders of this type that cannot be controlled by medicinal products and might benefit from the use of MDMS, as already shown by a certain number of well-designed and randomized studies of efficacy published in international medical literature. On the other hand, the continuous scientific advances in therapy over the last decades have allowed us to cure definitely important and severe diseases in the area of gastroenterology and hepatology. In order to understand better the difference between traditional medicinal compounds and MDMS in our specialty, it would be useful to show some of the excellent results obtained with the former drugs and the potential therapeutic opportunities of the latter in several conditions poorly controlled by pharmacological agents.

## 2 The revolutionary discoveries in the field of gastroenterology

### 2.1 Antisecretory drugs

The better knowledge of the physiological regulation of acid secretion by the gastric parietal cell has led to develop powerful pharmacological agents able to block the production of acid and to control the so-called acid-related diseases, mainly peptic ulcer and gastroesophageal reflux disease (GERD).

The development in the 1970s of H_2_-receptor antagonists provided incontrovertible evidence for the importance of endogenous histamine in the physiological control of gastric acid secretion (Black et al., 1972) and transformed the treatment of the above-mentioned diseases of the upper digestive tract. These drugs inhibit gastric acid secretion elicited by histamine in a dose-dependent manner and their action is maximal in basal (fasting) and nocturnal acid secretion (Savarino et al., 1988).

Later, at the end of 1980s, more powerful antisecretory agents were synthesized, the inhibitors of H + K + -ATPase (PPIs), which is the proton pump located in the apical membrane of the parietal cell and is the ultimate mediator of gastric acid secretion (Shin and Sachs, 2009). Also the pharmacological effect of PPIs is dose-related, but is more evident during the daytime, when acid secretion is stimulated by meals than during the nocturnal periods (Savarino et al., 1998). The duration of acid inhibition is longer-lasting than that of H_2_-blockers and this results in a more effective healing of both peptic ulcer and reflux esophagitis (Walan et al., 1989; Savarino et al., 2009).

More recently, a new class of antisecretory drugs, the potassium competitive acid blockers (pCABs), has been introduced into the market and they are characterized by a better pharmacokinetic and pharmacodynamic profile than PPIs (rapid onset of action, longer-lasting acid suppression and better control of nocturnal acidity) (Savarino et al., 2022). They are not pro-drugs that need to be activated in the canaliculi of oxyntic cells, like PPIs, and therefore their effect is rapid and evident within the first day of administration (Sakurai et al., 2015). Their antisecretory action relies on the inhibition of the proton pump by reducing K+ availability for the enzyme, which is essential for the maintenance of acid secretion (Abdel-Aziz et al., 2021).

From a clinical point of view, pCABs have the potential to improve the management of patients with acid-related disorders, including peptic ulcer and GERD because of their ability to overcome several important drawbacks of PPIs (slow onset of action, the scant effect on nocturnal acidity) and their action is not affected by genetic polymorphism, especially in Asian populations (Martinucci et al., 2017).

Overall, all the antisecretory agents developed in the last decades have a specific pharmacological action on a single target able to change the function of the gastric parietal cell in the production of acid and this has allowed us to cure the diseases due to acid hypersecretion and to subtract thousands of patients to the need for surgical therapy.

### 2.2 *Helicobacter pylori* infection

The discovery of *H. pylori* infection in the stomach has led to a major revolution in the science and practice of gastroenterology (Malfertheiner et al., 2022). Many studies have clearly shown that this germ is the major cause of gastritis and peptic ulcer, which have to be considered as infectious diseases that must be cured with antibiotics and no more with acid suppressants (Fock et al., 2013).

The most compelling evidence that *H. pylori* was indeed the main cause of peptic ulcer disease came from clinical trials because the eradication of the bacterium resulted in resolution of this disease that does not recur and is no more associated with dangerous complications, such as bleeding and perforation (Hopkins et al., 1996). Single agent therapy has proven ineffective *in vivo* and has led to the emergence of resistant strains, while double antibiotic therapy in combination with antisecretory drugs (triple therapy) has resulted successful to heal peptic ulcer (Bazzoli, 1996). Amoxicillin and clarithromycin have been the mostly recommended antibiotics in various eradication regimens (Mégraud, 1995). The former is a penicillinase-susceptible semi-synthetic penicillin and its antimicrobial activity consists in the inhibition of the cell wall transpeptidase and is bactericidal, while the latter is usually bacteriostatic and inhibits protein synthesis by binding reversibly to the 50S ribosomal subunit of sensitive micro-organisms (al-Assi et al., 1994).

Overall, the specific action of the two antibiotics on the essential structures of the bacterium with the block of their replication is the single mechanism responsible for the success of anti-Helicobacter therapy.

### 2.3 Chronic hepatitis C

Finally, an additional example of revolutionary therapeutic success is the development of powerful antiviral drugs, which have allowed us to cure a dangerous and severe disease, such as chronic hepatitis C, which is able to evolve to cirrhosis and hepatocarcinoma in many cases (Huang and Yu, 2020). In fact, the virus responsible for this disease (hepatitis C virus = HCV) can be eradicated virtually in all patients with short courses of the new direct-acting anti-viral agents (DAAs), generally from 6 to 24 weeks (Chhatwal et al., 2015).

The selection of the most convenient DAA regimen is firstly driven by the HCV genotype. If it is true that HCV elimination is immediately associated with liver improvements, halting and reversing hepatic fibrosis, also direct extra-hepatic complications of HCV replication, including mixed cryoglobulinaemia vasculitis, resolve after HCV eradication in most cases (Soriano et al., 2016). In addition, indirect extra-hepatic damage as result of persistent systemic inflammation ameliorates following HCV cure with improvements in diabetes, dyslipidemia and fatigue, along with a reduced incidence of cardiovascular events, renal disease and lymphomas (Negro and Hepatitis, 2013).

For instance, (Foster et al., 2016), using sofosbuvir combined with velpatasir, were able to eradicate HCV in more than 90% of their patients with HCV genotype 3 infection, which is the most difficult form to cure, and obtained 100% eradication in patients with the other genotypes after 12 weeks of treatment.

Once again, these excellent results in the therapy of HCV infection were due to the clinical use of DAAs, which are antiviral drugs acting as protease or polymerase inhibitors and then permit to block HCV replication with a single and precise mechanism of action (Ford et al., 2014).

## 3 The large body of functional disorders in gastroenterology

If the use of revolutionary pharmacological agents has allowed us to change dramatically the natural course of some important digestive and hepatic diseases, such as GERD, peptic ulcer and chronic hepatitis C, there are several other illnesses that affect many patients in the field of gastroenterology. They do not have a structural basis to explain their clinical features, which are generated by a complex interaction among various factors such as microbial dysbiosis within the gut, altered mucosal immune function, altered gut signaling (visceral hypersensitivity) and central nervous system dysregulation of the modulation of gut signaling and motor function (Drossman, 2016). They represent the most common diagnoses in gastroenterological units, their natural course tends to be chronic with alternate phases of remission and exacerbation, their prognosis is good because of the lack of anatomic involvement and therefore their major clinical consequence consists in reducing, more or less, the quality of life of patients.

It is evident that the complexity of their pathophysiological alterations, including motility disturbance, visceral hypersensitivity, altered mucosal and immune function, altered gut microbiota, and altered central nervous processing, cannot benefit from a single pharmacological agent with an exclusive effect on a specific target. Instead, therapeutic substances with non-pharmacological properties, but able to act by affecting or modulating different pathogenetic features, even though with unknown mechanisms of action, like MDMS, might have a role in alleviating symptoms of patients with functional gastrointestinal disorders. This potential effect has been already substantiated by many clinical trials performed with the clinical trial standards adopted for traditional medicinal products and therefore both their efficacy and safety have been objectively evaluated in the interest of patients undergoing this kind of treatment.

### 3.1 Esophageal disorders

Functional esophageal disorders present with typical symptoms (mainly heartburn and regurgitation) that are not associated with structural, inflammatory or major motor abnormalities. Thus, these patients have a normal endoscopy and no evidence of both eosinophilic esophagitis or achalasia and esophageal spasm or abnormal esophageal acid exposure. According to Rome IV criteria (Aziz et al., 2016), they are mainly represented by functional chest pain, reflux hypersensitivity and functional heartburn. The majority of these patients do not respond to PPI therapy because the pathogenetic role of acid is absent or very poor in them (Savarino et al., 2013a).

The pathophysiology of these disorders is complex and still unclear, but different factors seem to be implicated. It has been hypothesized that there is a combination of peripheral and central factors that interplay to increase esophageal perception. An increased permeability of esophageal mucosa due to an altered integrity with dilation of intercellular spaces (Savarino et al., 2013b) may allow noxious sensitizing luminal substances access to deeper layers of the esophagus, where they may induce an inflammatory response; then signals are transferred *via* spinal cord to the brain and an abnormal central processing may contribute to generate symptoms, in particular heartburn. Psychological factors, such as anxiety, may enhance the perception of peripheral stimuli of chemoreceptors and mechanoreceptors.

Given that abnormal peripheral sensitization and central processing are considered relevant in the pathogenesis of esophageal functional disorders, the treatment remains empiric and the use of pain modulators is strongly suggested (Fass et al., 2021). However, clinical trials aimed at assessing the efficacy of these drugs are scarce and disappointing (Savarino et al., 2020). On the contrary, there is some evidence that MDMS may be helpful in these conditions.

### 3.2 Examples of randomized and controlled studies with MDMS

Table 1 reports the most important features of the placebo-controlled clinical trials we have evaluated in the field of esophageal functional disorders.

**TABLE 1:** Randomized and controlled studies with MDMS in esophageal functional disorders.

Authors, year	Types of disorder	No. of cases	Types of intervention	Results
Palmieri et al. (2013)	NERD	20	HA + CS vs. placebo x 2 weeks	Significantly higher relief of symptoms with MDMS (*p* < 0.01)
Savarino et al. (2017)	NERD	154	HA + CS + PPI vs. PPI + placebo x 2 weeks	Significant improvement of reflux symptoms with MDMS + PPI (*p* < 0.01)
Ribaldone et al. (2021)	NERD	20	HA + aminoacid mixture and rice extract vs. placebo x 2 weeks	Significantly higher relief of reflux symptoms with MDMS (*p* < 0.0001)
Pellegatta et al. (2022)	GERD with extraesophageal symptoms	71	HA + CS + aloe and honey combined with PPI vs. PPI alone x 6 weeks	No statistical difference between the two treatments on the relief of atypical symptoms (*p* > 0.05)

Abbreviations: NERD, non-erosive reflux disease; HA, hyaluronic acid; CS, chondroitin sulphate; MDMS, medical device made of subctances; PPI, proton pump inhibitors; GERD, gastroesophageal reflux disease.

Among the MDMS available for the treatment of functional esophageal disorders, one of the most important and most studied is represented by the combination of hyaluronic acid (HA), which is well known for its regeneration properties and tissue repair, and chondroitin sulphate (CS), which has an anti-inflammatory and mucosal protective activity (Savarino et al., 2017). These two substances are linked to poloxamer 407, which provides high adhesive properties and permits to prolong the contact time with esophageal mucosa. This compound acts as a physical barrier and not as a chemical agent.

(Di Simone et al., 2012) performed an experimental study on the esophageal mucosa of pigs and, using Evans blue as a dye which appears when there is an increased mucosal permeability, they showed that there is no stain when the above compound was added to an acid damaging solution given for 90 min, thus confirming its very high esophageal protective effect.

From a clinical point of view, (Palmieri et al., 2013) studied a small group of 20 patients with typical symptoms of non-erosive reflux disease (NERD), that is heartburn and regurgitation. They received four daily doses of HA + CS for 2 weeks and placebo with a cross-over design. The authors found that this combination relieved symptoms significantly more than placebo (*p* < 0.01).

Later, our group (Savarino et al., 2017) performed a larger study enrolling 154 patients with NERD. They were subdivided into two groups receiving HA + CS and PPIs or PPIs + placebo. One dose of PPIs and four daily doses of HA + CS and placebo were given each day for 2 weeks. HA + CS added to PPIs was able to reach the primary end point, that is the reduction of the total symptom score by at least three points, in 52% of patients compared with 32% of PPIs + placebo and this difference was highly significant (*p* < 0,01). Also the quality of life evaluated with the SF-36 questionnaire resulted to be improved with the former combination, in particular general health and social functioning. Therefore, HA + CS are able to ameliorate both reflux symptoms and quality of life in NERD patients, when given both alone or associated with PPIs in comparative studies with placebo. The adverse events recorder during the study resulted to be similar in patients treated with MDMS and placebo.

Another small study by (Ribaldone et al., 2021) assessed the efficacy and safety of a patented oral formulation (liquid sachets containing HA + *a* mixture of amino-acids including proline, hydroxy-proline and glutamine, and rice extract dispersed in bio-adhesive polymer matrix) in a randomized, double-blind and placebo-controlled study enrolling 20 NERD patients with heartburn, who were treated with three sachets per day for 2 weeks. The authors found that a three-point reduction in the total symptom score was achieved in 95% of patients with the investigational product against 20% of patients with placebo (*p* < 0,0001). No adverse events were reported.

Finally, (Pellegatta et al., 2022) evaluated the efficacy of a 6-week treatment with a MDMS consisting of HA + CS and aloe + honey combined with PPI against PPI monotherapy in 71 patients with extra-esophageal symptoms of GERD. The comparison between groups did not show statistically significant differences, while the combined product was significantly superior to PPI alone for individual items of the total Reflux Symptom Index (RSI). This is the first published study on the effects of a new MDMS in the treatment of a difficult condition, such as GERD presenting with extra-esophageal symptoms. Only minor adverse events have been documented in this clinical study.

## 4 Functional dyspepsia

At least 20% of the general population has chronic recurrent symptoms that can be attributed to disorders of gastroduodenal function and these people do not have any evidence of organic causes (Tack et al., 2006). Functional dyspepsia (FD) is characterized by one or more of the following symptoms: Post-prandial fullness, early satiation, epigastric pain and epigastric burning. Two groups of patients have been identified: Patients with post-prandial distress syndrome (PDS), which is characterized by meal-induced dyspeptic symptoms and epigastric pain syndrome (EPS), which refers to symptoms that do not occur exclusively post-prandially, but can also occur during fasting. In an additional group, the two syndromes can overlap (Stanghellini et al., 2016).

The pathophysiology of FD is complex and multifactorial, like the one showed for esophageal functional disorders. It is far from being elucidated and comprises gastroduodenal motor and sensory dysfunction, as well as impaired mucosal integrity, low-grade immune activation and dysregulation of the gut-brain axis. Acid hypersecretion is not implicated in the pathogenesis of FD (Savarino et al., 2011) and also H. pylori infection has a modest role (Blum et al., 1998). The association of dyspepsia and psychiatric disorders, such as anxiety, depression and neuroticism, is commonly recognized (Henningsen et al., 2003).

Treatment is empiric and generally addressed to control at least one of the pathophysiological factors sustaining the most disturbing symptoms. Although many clinical trials using the traditional pharmacological agents (PPIs, prokinetics, anti-Helicobacter antibiotic regimens, pain modulators, etc.,) have been made, the results have been frequently partial and unsatisfactory (Talley, 1991). Even the adoption of psychological therapies has not provided convincing benefit because of the small sample sizes and poorly matched treatment groups (Stanghellini et al., 2016).

### 4.1 Examples of randomized and controlled studies with MDMS

Overall, it is not surprising that no single pharmacological agent was shown to control adequately the complex pathophysiological alterations of FD and therefore these patients continue to suffer from their symptoms even for the entire life, whose quality is greatly reduced. However, several randomized and controlled clinical trials using MDMS have been published in recent years and showed promising results. They are displayed in Table 2.

**TABLE 2:** Randomized and controlled studies with MDMS in patients with functional dyspepsia.

Authors, year	Type of disorder	No. of cases	Types of intervention	Results
Chey et al. (2019)	Functional dyspepsia	95	Caraway oil + L menthol vs. placebo for 2 weeks	Significantly higher reduction of symptoms with MDMS (*p* < 0.039)
Rich et al. (2017)	Functional dyspepsia	114	Peppermint + caraway oil vs. placebo x 4 weeks	Significantly higher relief of symptoms with MDMS (*p* < 0.001)

Abbreviation: MDMS, medical device made of substances.

(Chey et al., 2019) conducted a randomized and controlled trial, which evaluated a novel formulation of caraway oil and L-menthol *versus* placebo in patients with FD defined by Rome III criteria (Tack et al., 2006). Ninety-five patients were randomized to receive the investigational product (two capsules per dose, twice per day) or placebo and efficacy was measured at 24 h, 2 weeks, and 4 weeks. At 24 h, the active arm reported a statistically significant reduction in PDS symptoms (*p* < 0.039). In patients with more severe symptoms, approximately 3/quarter of them showed substantial global improvement for those with EPS syndrome (*p* < 0.046) after 4 weeks of treatment against half in the control arm. Overall, this study showed that the combination of caraway oil and L-menthol was able to provide rapid resolution of symptoms (within 24 h) and to control severe FD with EPS symptoms after 4 weeks of treatment compared with placebo.

In an additional study (Rich et al., 2017) assessed the efficacy of a fixed peppermint/caraway oil combination (Menthacarin) on symptoms and quality of life of patients with FD (both PDS and EPS), performing a prospective, double-blind trial with 114 outpatients who were randomized to receive this compound or placebo (2 × 1 capsule/day) for 4 weeks. After 2 and 4 weeks, active treatment was superior to placebo in alleviating symptoms in both forms of dyspepsia (*p* < 0.001). The authors concluded that Menthacarin is an effective therapy for the relief of pain and discomfort and the improvement of the quality of life in patients with FD, suffering from both EPS and PDS forms.

## 5 Functional bowel disorders

They are a spectrum of chronic gastrointestinal disorders characterized by predominant symptoms of abdominal pain, bloating, distension and/or bowel habits abnormalities (constipation, diarrhea, or mixed constipation and diarrhea). These disorders are not due to anatomic abnormalities identified by routine diagnostic examinations. The main categories are the following: Irritable bowel syndrome (IBS), functional constipation (FC), and functional diarrhea (FDr) (Longstreth et al., 2006).

### 5.1 Irritable bowel syndrome

IBS is characterized by recurrent abdominal pain combined with change in bowel habits (constipation, diarrhea or mixed constipation and diarrhea). There are frequently associated symptoms of abdominal bloating/distension. The pathophysiology is complex and multifactorial and includes altered gastrointestinal motility, visceral hypersensitivity, increased intestinal permeability, prior enteric infections, immune activation, altered microbiota, and disturbancies in brain-gut interaction (Mearin et al., 2016). Moreover, the presence of psychological alterations is frequent in IBS patients and may interplay with the above multiple factors. Treatment is based on the control of the mostly disturbing symptoms and therefore many pharmacological agents are usually adopted, but the benefit is limited and the natural history of the disease does not change.

### 5.2 Functional constipation

This is a disorder in which symptoms of difficult, infrequent or incomplete defecation predominate, while abdominal pain is absent or minimal. Similar to IBS, it is due to a variety of pathophysiological processes and moreover psychological factors may be frequently associated. Medical therapy is empiric and based on different options that do not modify the course of the disorder which remains chronic and recurrent.

### 5.3 Functional diarrhea

This form is characterized by recurrent passage of loose or watery stools and this is the predominant symptom, while abdominal pain is generally lacking or poorly represented. Once again, there is no single pathophysiological alteration capable to explain the cause of symptoms and, like IBS, various mechanisms contribute to generate the clinical manifestations of the disorder, including both physiologic and psychosocial factors. Treatment is addressed to control the main symptom diarrhea with several pharmacological products, which however are not able to cure definitely the disease.

### 5.4 Examples of randomized and controlled studies with MDMS

As above-mentioned, patients with functional bowel disorders are treated with a wide variety of drugs and forms of psychotherapy, but the multiplicity of therapy proves that none is strikingly effective, an observation made daily by clinicians caring for these patients (Klein, 1988). Many factors are implicated in symptom generation and therefore a single agent does not provide notable enduring success. For these reasons the use of MDMS may be helpful in reaching good therapeutic results and many clinical trials have already shown that they are effective and safe in many patients. Particularly those with FC, both children and adults, have been the object of multiple studies and various meta-analyses, as shown in Table 3.

**TABLE 3:** Randomized and controlled studies with MDMS in patients with functional bowel disorders.

Authors, year	Types of disorder	No. of cases	Types of intervention	Results
Strisciuglio et al. (2021)	Functional constipation in children	158	Complex of honeys + aloe and Mallow polysaccharides (Promelaxin) microenemas vs. polyethylene glycol (PEG) 4,000 × 2 weeks	Non-inferiority of Promelaxin microenemas compared with PEG 4000 in alleviating constipation
Minguez et al., 2016	Functional constipation in adults	1,099	Systematic review of 12 studies comparing PEG vs. placebo	Significant increase in stool number with PEG
Lee-Robichaud et al. (2010)	Functional constipation in adults and children	868	Meta-analysis of 10 studies (RCTs) comparing PEG vs. lactulose	PEG was better than lactulose in outcomes of stool frequency, form of stools and relief of abdominal pain
Gordon et al. (2016)	Functional constipation in children	101 (PEG vs. placebo)	Meta-analysis of 2 RCTs in children FC comparing PEG vs. placebo, 6 RCTs comparing PEG vs. lactulose and 3 RCTs comparing PEG vs. milk of magnesia	Significantly increased number of stools per week with PEG compared with placebo, lactulose and milk of magnesia, respectively
		465 (PEG vs. lactulose)		
		211 (PEG vs. milk of magnesia)		

Abbreviations: MDMS, medical device made of substances; PEG, polyethylene glycol; RCT, randomized controlled trial; FC, functional constipation.

(Strisciuglio et al., 2021) conducted a randomized non-inferiority trial in order to assess whether microenemas of Promelaxin (A complex of honeys + Aloe and Mallow polysaccharides) are not inferior to oral polyethylene glycol (PEG) 4,000 as topical therapy in children with FC according to Rome III criteria (Longstreth et al., 2006). They enrolled infants and young children aged 6–48 months who were randomized to 2 weeks of Promelaxin microenemas or PEG daily, followed by a 6-week on-demand treatment period. The primary endpoint was defined as to achieve at least three evacuations per week and an average increase of at least one evacuation per week as compared to baseline. One hundred and fifty-eight patients were recruited and the study showed that Promelaxin in microenemas was not inferior to PEG 4,000 in reaching the primary objective (response rate difference: 16,5%, CI 1.55%−31,49%, with Promelaxin *versus* 11,03%, CI 5.58%–27,64%) with PEG.

A recent review (Mínguez et al., 2016) has evaluated the evidence published on the use of PEG, with or without electrolytes, against placebo in the management of FC and found that all studies showed a significant superiority regarding stool number, less straining, less need for rescue laxatives and lower dropout number in patients taking PEG. Remarkable secondary effects were not observed.

In a Cochrane review from 2010, (Lee-Robichaud et al., 2010) performed a meta-analysis of clinical trials published between 1997 and 2007 that comparatively evaluated PEG solutions with lactulose for FC treatment. They included ten trials in the review with a total of 868 patients and concluded that PEG is superior to lactulose regarding the increment in the number of stool passages/week, form of the stool, decrease in bowel pain and reduction in the use of associated laxatives.

Finally, (Gordon et al., 2016) assessed the efficacy and safety of osmotic laxatives used to treat FC in children and concluded that PEG preparations were superior to placebo, lactulose and milk of magnesia in terms of increased number of stools per week. The adverse events included flatulence, abdominal pain, nausea, diarrhea and headache.

## 6 Discussion and conclusion

In the last decades we have witnessed revolutionary improvements in the science and therapy of several gastroenterological diseases that have been controlled adequately or cured definitively. They are represented by the so-called acid-related diseases, in particular GERD, and by two relevant infectious illnesses, such as peptic ulcer and chronic hepatitis C, which have been resolved with the use of antimicrobial or antiviral agents acting on single pharmacological targets able to block the replication of both *H. pylori* or HCV.

However, pharmacological agents have remarkable limitations when adopted to cure patients with other digestive disorders, which are characterized by multifactorial and not fully elucidated pathophysiological alterations that are difficult to treat with traditional drugs whose effect is based on a specific and precise mechanism of action. These disorders affect the entire gastroenteric tract, are very frequent and present a chronic and recurrent natural history. Due to their complex pathogenesis and the absence of anatomical abnormalities, they are named functional disorders because of the supposed dysfunction of the brain-gut interaction in the generation of their symptoms.

These patients with gastrointestinal functional disorders are the most suitable candidate for the use of MDMS in the field of gastroenterology. Indeed, the pathophysiological mechanisms inducing these widespread clinical conditions are complex and poorly understood and therefore they do not benefit from pharmacological agents acting on a single target. The use of MDMS, whose mechanism of action is not pharmacological, but may be linked to a multiple synergistic effect on many different factors, can control better the symptoms of these patients. A medical device made of complex or natural substances and devoid of a single target effect can profitably and synergistically act on the multiple factors implicated in the pathogenesis of these diseases. The non-pharmacological effect of MDMS is favored by the fact that the digestive tract has the fundamental function of being a barrier that can be reinforced physically by the use of agents with non-specific mechanism of action.

There are already many clinical trials, performed with the well-known standards of randomized and controlled studies, that seem to confirm their efficacy and safety in the treatment of the various functional illnesses pertaining to the gastroenterological world. So far, the controlled clinical studies performed and evaluated in our paper have confirmed that MDMS are safe and the adverse events registered in the various RCTs are of minor severity and superimposable to those of placebo. Obviously, the post-marketing surveillance plays a central role in collecting and managing any report on adverse events and reactions regarding these products.
